# Insights into Minor Group Rhinovirus Uncoating: The X-ray Structure of the HRV2 Empty Capsid

**DOI:** 10.1371/journal.ppat.1002473

**Published:** 2012-01-05

**Authors:** Damià Garriga, Angela Pickl-Herk, Daniel Luque, Jürgen Wruss, José R. Castón, Dieter Blaas, Núria Verdaguer

**Affiliations:** 1 Institut de Biologia Molecular de Barcelona (CSIC), Parc Científic de Barcelona, Barcelona, Spain; 2 Department of Medical Biochemistry, Max F. Perutz Laboratories, Vienna Biocenter, Medical University of Vienna, Vienna, Austria; 3 Centro Nacional de Biotecnología (CSIC), Cantoblanco, Madrid, Spain; Institut Pasteur, France

## Abstract

Upon attachment to their respective receptor, human rhinoviruses (HRVs) are internalized into the host cell via different pathways but undergo similar structural changes. This ultimately results in the delivery of the viral RNA into the cytoplasm for replication. To improve our understanding of the conformational modifications associated with the release of the viral genome, we have determined the X-ray structure at 3.0 Å resolution of the end-stage of HRV2 uncoating, the empty capsid. The structure shows important conformational changes in the capsid protomer. In particular, a hinge movement around the hydrophobic pocket of VP1 allows a coordinated shift of VP2 and VP3. This overall displacement forces a reorganization of the inter-protomer interfaces, resulting in a particle expansion and in the opening of new channels in the capsid core. These new breaches in the capsid, opening one at the base of the canyon and the second at the particle two-fold axes, might act as gates for the externalization of the VP1 N-terminus and the extrusion of the viral RNA, respectively. The structural comparison between native and empty HRV2 particles unveils a number of pH-sensitive amino acid residues, conserved in rhinoviruses, which participate in the structural rearrangements involved in the uncoating process.

## Author Summary

Human Rhinoviruses (HRVs), members of the *Picornaviridae* family, are small non-enveloped viruses possessing an icosahedral capsid that protects the single-stranded RNA genome. Although much is known about their binding to cell receptors and their uptake into the host cell, the mechanism by which their genomic RNA leaves the capsid and arrives to the cytosol to initiate replication is poorly understood. In HRV2, a member of the minor group HRVs, upon binding to lipoprotein receptors (LDL-R) on the cell surface virions are taken up into vesicles and directed to early endosomes. The low pH conditions found in the endosome, and not the binding to LDL-R, catalyze the delivery of the viral genome. The crystal structure of the HRV2 empty particle, representing the last stage of the uncoating process, unveils the structural rearrangements produced in the viral capsid during the externalization of the VP1 N-terminus and the delivery of the genomic RNA. We propose that RNA exit occurs through large capsid disruptions that are produced at the particle two-fold symmetry axes. Our data also suggests that the VP1 N-terminus would be externalized through a new pore, opening at the canyon floor.

## Introduction

A key step in the life cycle of viruses is the delivery of its genome into a compartment of the host cell appropriate for its replication. This involves the recognition of specific cell surface receptors by the viral capsid and the passage of the genome across at least one membrane barrier. Enveloped viruses achieve this by fusing with cellular membranes. In non-enveloped viruses, including picornaviruses, the capsid proteins and the viral capsid as a whole must provide the machinery for the translocation of the viral genome in a process that remains poorly understood. The discovery of short, membrane altering amphipathic or hydrophobic sequences in capsid proteins, such as the VP1-N-terminus of poliovirus (PV) [1] and the entire VP4 in human rhinovirus 16 [2], suggested that these peptides were involved in breaching host membranes. While crystallographic structures of various picornaviruses showed that VP4 and the N-terminus of VP1 line the interior of the capsid, biochemical experiments revealed that these peptides can be transiently exposed, demonstrating that the virions are highly dynamic and temporarily externalize otherwise internal structures via ‘breathing’ [3], [4].

Human rhinoviruses (HRVs), members of the *Picornaviridae* family, are the cause of about 50% of all mild infections of the upper respiratory tract. Despite being rarely life threatening, prevalence and recurrent nature of the common cold make these viruses of paramount economic importance due to the huge expenditures for medication and working days lost. Based on the complete genome sequences, HRVs were classified as three species within the genus *Enteroviruses* that included 74 HRV-A, 25 HRV-B and 7 HRV-C [5]. Independent from phylogeny, HRVs are also classified on the basis of receptor usage: the minor receptor group, 12 HRV-A, bind low-density lipoprotein receptor (LDLR), very-LDLR (VLDLR) and LDLR-related protein (LRP) [6], while the remaining HRV-A and HRV-B, which belong to the major receptor group, use intercellular adhesion molecule 1 (ICAM-1) for cell entry [7]. Some major group HRVs might also use heparan sulphate as an additional receptor either with or without adaptation in tissue culture [8]-[10]. The receptor(s) binding the recently identified HRV-Cs are still unknown [11]. A characteristic trait of all HRVs is their instability at low pH, which distinguishes them from the otherwise closely related enterovirus species A to D, including the 3 polioviruses.

Like all members of the *Picornaviridae family*, HRVs consist of a T = 1 (pseudo T = 3) icosahedral capsid of about 30 nm in diameter that is built from 60 copies of each of 4 coat proteins VP1, VP2, VP3, and VP4, protecting a plus-sense single-stranded RNA genome. A prominent feature of the HRV shell is a star-shaped mesa on each of the five-fold symmetry axes that, in the case of minor group HRVs, harbors the binding sites for the LDL family of receptors [12]–[14]. The five-fold vertex is surrounded by a large depression or canyon, containing the binding site for the ICAM-1 receptor in major group HRVs [15], [16]. At the bottom of the canyon, buried between the two ß-sheets of the VP1 core, there is a hydrophobic pocket which in some picornavirus capsid structures is occupied by elongated electron densities that have been modeled as different fatty acid cofactors, known as pocket factors. These molecules are thought to stabilize the native conformation of the virions [17]–[23]. A number of drugs with antiviral activity have been shown to bind in this pocket, displacing the pocket factor because of their higher binding affinity. Antiviral drugs bound to the pocket rigidify the capsid, preventing the required structural changes [3], [24]–[26]. The stabilizing role of the pocket factor has been challenged by more recent data on HRV14, showing that mutations filling the VP1 pocket have no effect on viral replication. It has been suggested that the VP1 pocket itself regulates the structural dynamics required for viral infection [27].

Although much is known about the binding of rhinoviruses to their receptors and their uptake into the cell, the mechanism by which their genomic RNA leaves the capsid, crosses the endosomal membrane and arrives to the cytosol is still enigmatic. The different dependence on receptor function and pH for initiation of RNA release in the various HRV serotypes adds an additional dimension to this problem [28]. In major group HRVs and PVs, interaction with the receptor initiates irreversible structural changes and exit of VP4. In contrast, in the minor group viruses the low endosomal pH alone triggers these changes [29] and the receptor might rather have a stabilizing function [30], [31].

The subviral A-particles, remaining after loss of VP4, sediment at 135S (compared to 150S for the native virion). A-particles are the dominant form of the virus found in cells early in infection. They are believed to be a necessary intermediate in the entry process and, in the case of poliovirus, have been demonstrated to be infectious [32], [33]. With respect to native virions, A-particles exhibit changes in antigenicity and sensitivity towards protease digestion, have increased hydrophobicity and readily attach to liposomes [34], likely through the exposure of N-terminal residues of VP1 [4]. The N-terminal segment of VP1, possibly in conjunction with VP4, may facilitate RNA translocation into the cytoplasm by forming a pore in the endosomal membrane. This is supported by the observation that, at comparably higher concentrations, the derived peptides alone can permeabilize membranes [2], [35], [36].

After release of the RNA, empty capsids (B-particles) sedimenting at 80S appear and polyprotein synthesis commences [37]. Similar empty particles can be produced *in vitro* by exposure of native virions to pH≤5.6, or by incubation at 50 to 56°C in low ionic strength buffer [37], [38].

A body of experimental data from cryo EM studies [39]–[45] addressed the low and medium resolution structures of the subviral particles of PVs and HRVs. All of these studies showed significant alterations occurring concomitant with genome release: externalization of myristoyl-VP4 and the N-terminus of VP1, expansion of the virus particle and an iris-like movement extending the pores at the five-fold axes. In a widely accepted model, it was proposed that receptor binding to the viral capsid (and/or low pH) induced changes widening the channel at the five-fold axes, allowing for VP4, the N-terminus of VP1 and the viral RNA to exit the capsid [16], [39], [46], [47]. A number of cryo-EM structures were also compatible with an alternative model in which VP4 and the N-termini of VP1 exit via pores at the base of the canyon and the RNA egresses via the channel at the five-fold axis channel [40], [41], [43], [44]. A recent work in PV provided additional evidences of the site of egress of VP1 at the base of the canyon by locating the binding site for the Fab fragment of an antipeptide antibody directed against the VP1 N-terminus [48]. Furthermore, new cryoEM studies of the PV 80S particles, together with the cryo-electron tomography characterization of an additional state, in which the particles were caught in the act of RNA release, suggested that the viral RNA exits through holes close to the two-fold axes not far from the site at which N-terminus of VP1 exits in 135S particles [42], [45].

However, the low and medium resolution of the cryo-EM reconstructions were insufficient to establish neither the detailed structural changes occurred during the formation of the subviral particles nor the identification of the specific interactions involved in the stabilization of these changes. Also, until now, crystallization of subviral particles had not been achieved. Here we report the 3.0 Å resolution crystal structure of the HRV2 empty particle. This structure and its comparison with the native virus shed light on the structural rearrangements produced in the viral capsid during RNA uncoating, unveiling the interactions involved in the pH-triggered conformational changes.

## Results

### Overall Structure of the HRV2 80S Empty Capsid

The crystal structure of the HRV2 80S empty particle was determined at 3.0 Å resolution by molecular replacement using 15-fold non-crystallographic symmetry averaging, starting with the phases corresponding to the native HRV2 structure [18]. The resulting averaged maps showed well-defined density and allowed rebuilding of most of the conformational changes that had occurred on transition from native HRV2 capsid to the 80S B-particle. Analysis of the electron density confirmed the absence of VP4, as predicted from comparison with the poliovirus uncoating scenario [44] and the cryo-EM structure of HRV2 previously determined at 15 Å [40]. Externalization of N-terminal residues of VP1 in the empty capsid is compatible with the lack of ordered electron density up to position 1062 (VP1, VP2 and VP3 proteins are numbered starting with 1000, 2000 and 3000 respectively).

**Figure 1:**
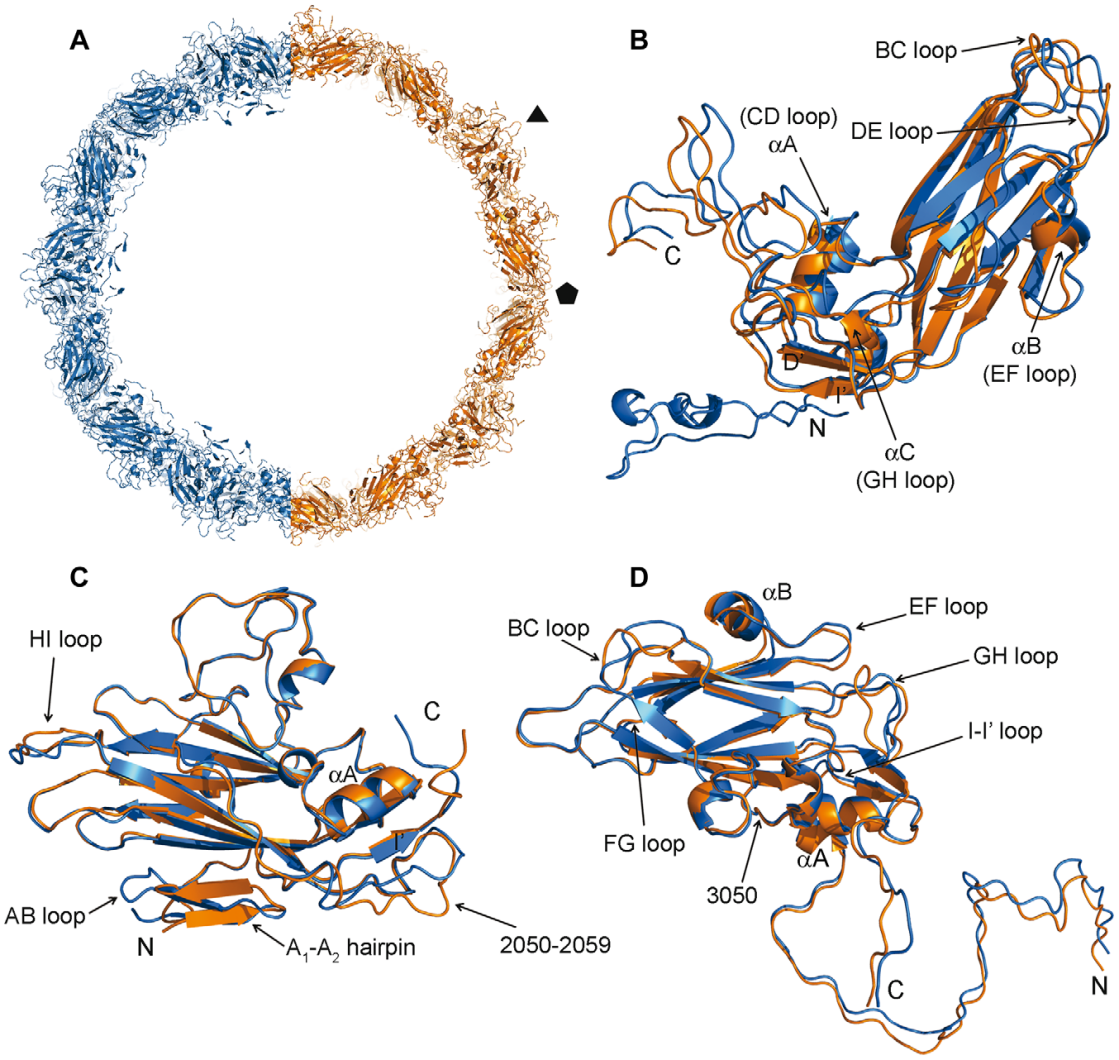
Overall comparison of native HRV2 and the derived B-particle. (A) Cartoon diagrams of the native (blue) and the 80S subviral B-particle (orange). Only half of each capsid shell is represented, as a ∼80 Å slab, to illustrate the expansion of the 80S particle with respect to the native virion. The positions of one three-fold and one five-fold axis of the icosahedral particle are indicated with black symbols. Superimposition of the β-barrels of VP1 (B), VP2 (C) and VP3 (D) capsid proteins in the two structures (native capsid in blue, 80S particle in orange). The structural motifs corresponding to the most relevant differences are indicated.

The overall icosahedral organization of the 80S particles is similar to that of the native virus. However, the 80S capsid has an average diameter of 326 Å (calculated using VIPERdb [49]). When compared with the corresponding 314 Å diameter of the native virions, this implies an average expansion of 3.8% (Figure 1A and Video S1), which is in accordance with the observations derived from electron microscopy studies for this virus and other related picornavirus particles [39]–[41], [43], [45], [50]. As the empty shell is derived from the native structure, this expansion implies a reduction in the thickness of the protein layer. Indeed, the average thickness of the HRV2 80S particle shell (measured as the difference between the averaged outer and inner radius obtained from VIPERdb) is 51 Å, in contrast to the 56 Å found in the native virion (Figure 1A and Video S1). Moreover, the 80S capsid appears smoother than the native capsid; on average, the canyon in the 80S particle is approximately 5 Å smaller in depth and width.

### Changes in the 80S Protomer

The overall shape of the protomer and the disposition of the VP1-3 subunits are maintained in the native, the 135S A-particles (Pickl-Herk et al., in preparation) and the 80S B-particles, despite of a number of changes have occurred. Structural comparisons of the native and the 80S protomers using SHP program [51] resulted in a root-mean-square deviation (r.m.s.d.) of 0.93 Å for the superimposition of 625 Cα atoms. The superimpositions of the individual VP1, VP2 and VP3 proteins in the native and the 80S structures gave r.m.s.d. values of 1.17 Å, 0.55 Å and 0.81 Å for the superimposition of 199, 219 and 212 equivalent Cα atoms, respectively. The biggest changes were concentrated in the N-terminal regions and in the loops connecting the strands of the ß-barrel. Thus, new superpositions were performed for each VP with program Lsqkab [52], using the ß-barrel core as a guide (Figures 1B–D).

**Figure 2:**
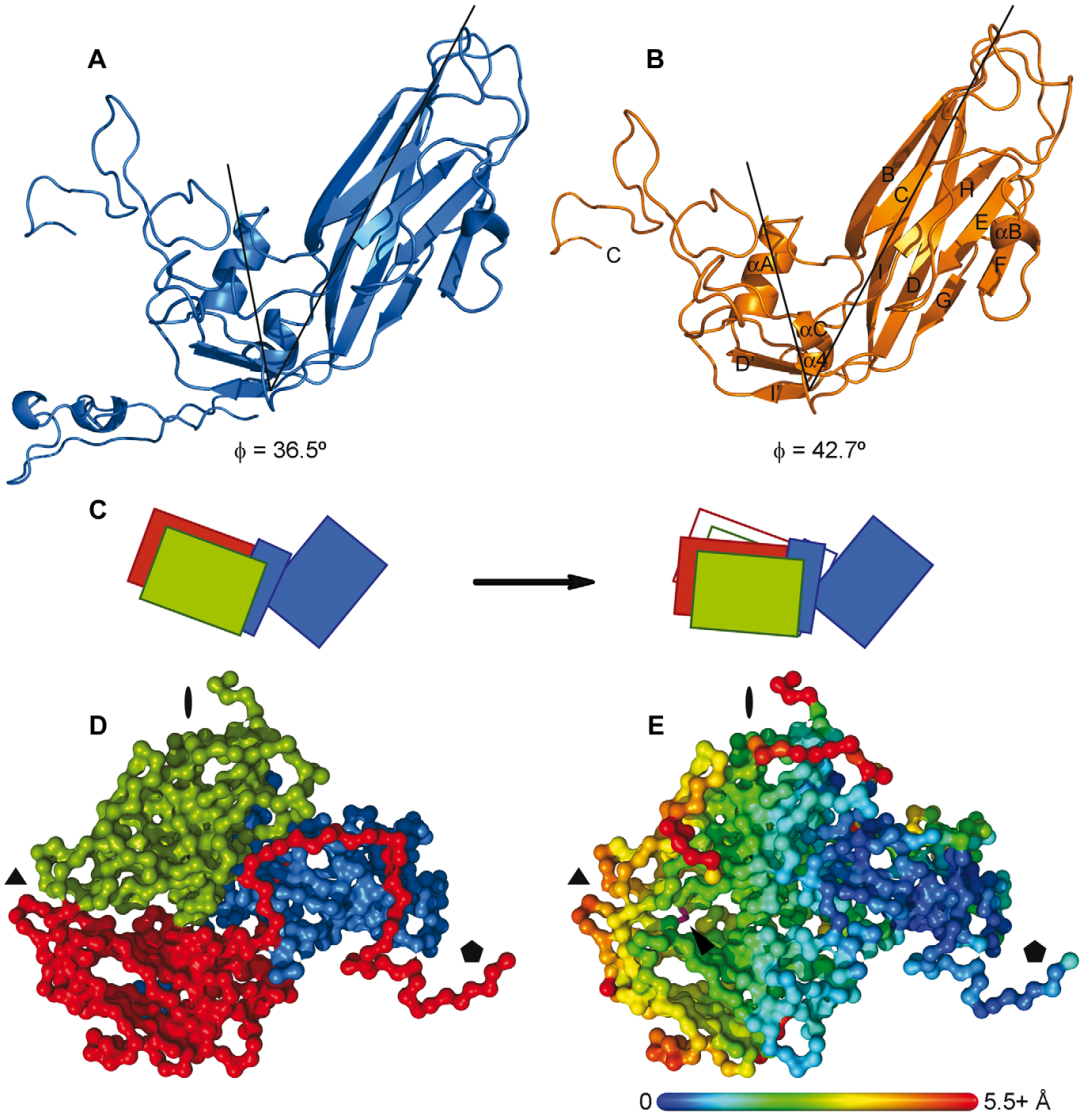
The hinge movement of VP1. Side by side comparison of the VP1 structures from the native virion (A) and the 80S particle (B), showing the hinge movement displacing the αA helix and the C-terminus of VP1 away from the VP1 β-barrel. The secondary structural elements are indicated on the 80S VP1 representation. The angle formed by the position of the Cα atoms from residues Ser1130, Lys1243 and Ala1105 and its value is also shown in both cases. (C) Schematic representation of the protomer expansion in the transition from the native virion (left) to the 80S particle (right). The hinge movement of VP1 (shown in blue) induces a concerted displacement of VP2 and VP3 (in green and red, respectively). To facilitate the comparison, the outer limits of the native protomer are also displayed in the 80S figure as thin lines. (D) Surface representation of the 80S protomer, seen from the interior of the particle, following the five-fold axis. VP1 is shown in blue, VP2 in green and VP3 in red. For clarity, only the Cα are displayed. The position of the symmetry axes is indicated with black symbols (E) Same view as in (D), with each residue colored according to the displacement suffered for its Cα in the transition from the native capsid to the 80S particle. The color scale, indicated as a bar, covers distances from 0 Å (in dark blue) to 5.5 Å or more (in red). The distances were calculated from a superposition of the native and the 80S protomers, using the VP1 β-barrel as a guide. The disulfide bridge linking Cys2229 and Cys3120 is shown as purple sticks and indicated with an arrowhead.

**VP1.** Superposition of the ß-barrels of the two VP1 structures allows the definition of two regions with different levels of resemblance: i) the ß-barrel and ii) the region comprising the CD loop, the GH loop and the C-terminal end of the protein (Figures 1B and 2). Breaking the VP1 molecule into these independent rigid bodies allowed the superimposition of 206 Cα atoms with a r.m.s. deviation of 0.76 Å. Thus, the rearrangement of VP1 in the 80S empty capsid with respect to the native virion can be explained by a hinge-type movement, consisting in an approximately 5.6° outward rotation of one of these two regions with respect to the other (Table S1). The pivot of this rotary motion is located in the ß-strand I' region (close to residue Lys1243) (Figures 1B, 2A and 2B).

The conformation of other loops has also changed to some extent: the EF-loop is displaced towards the base of the barrel and the short αB helix, contained within, is moved by about 3Å (Figure 1B); the BC loop at the five-fold axis appears highly flexible, as indicated by the highest B factors observed in the region (102.9 Å^2^) in comparison with the average (Table 1); the DE loop residues, from Asp1135 to Gly1137, also at the five-fold axis, are displaced upwards by 2.5 Å (Figure 1B).

**Table 1:** Data collection and refinement statistics.

Data collection	
Number of crystals	10
Wavelength (Å)	0.979/1.006
Resolution range (Å) (outermost shell)	60.7−3.0 (3.15−3.0)
Space group	I222
Cell dimensions	
*a*, *b, c* (Å)	a = 314.01, b = 356.85, c = 382.47
α = ß = γ (°)	90
Number of total/unique reflections	980753/308652
*R*_merge_ †	0.20 (0.45)
*I*/σ*I*	5.7 (1.4)
Completeness (%)	72.7 (40.3)
Redundancy	3.2 (1.3)

Refinement	
R*_factor_* ‡	0.26
No. of protein atoms	5515
Averaged B-factors (Å^2^)	50.62
R.m.s deviations: Bond lengths (Å)	0.007
R.m.s deviations: Bond angles (°)	1.4
Ramachandran plot	
Residues in preferred regions	643 (91.7%)
Residues in allowed regions	58 (8.3%)

**†:** *R*_merge_ = Σ*_hkl_*Σ*_i_*|*I_i_*(*hkl*)−<*I*(*hkl*)>|/Σ*_hkl_*Σ*_i_I_i_*(*hkl*), where *I* is the observed intensity and <*I*> is the average intensity of multiple observations of symmetry-related reflections.

**‡:** *R*_factor_ = Σ*_hkl_*|*F_obs_*(*hkl*) − *F_calc_*(*hkl*)|/Σ*_hkl_F_obs_*(*hkl*), where *F*obs and *F*calc are the structure factors, deduced from measured intensities and calculated from the model, respectively.

Finally, the N-terminal 61 residues of VP1 were not seen in our density maps, indicating that this region is disordered in the crystal structure of the 80S empty capsid. The first amino acid residue seen in the density (Arg1062) is located at the capsid interior, just below the canyon floor.

**VP2.** Superposition of the VP2 ß-barrels of the structures of native virus and 80S empty capsid also highlights the conservation of the overall folding, with only some local rearrangements (Figure 1C). In particular, the N-terminal ß-hairpin, which participates in the inter-pentameric contacts, is displaced by about 3 Å and twisted by 20° around the axis given by the ß-strand A_2_. The C-terminus also diverges from the native structure, especially from residue Ser2254 onwards, just after the strand I'. A number of rearrangements are also observed in loops connecting the ß-strands of the VP2 barrel; residues Leu2231-Thr2239 within the HI-loop, at the three-fold axis, are moved by more than 3 Å away from their position in the native structure. The loop preceding the B strand, including residues from Ile2050 to Ser2059, at the two-fold axis, seems to be highly flexible, with averaged B-factors of 127.8 A^2^.

**Figure 3:**
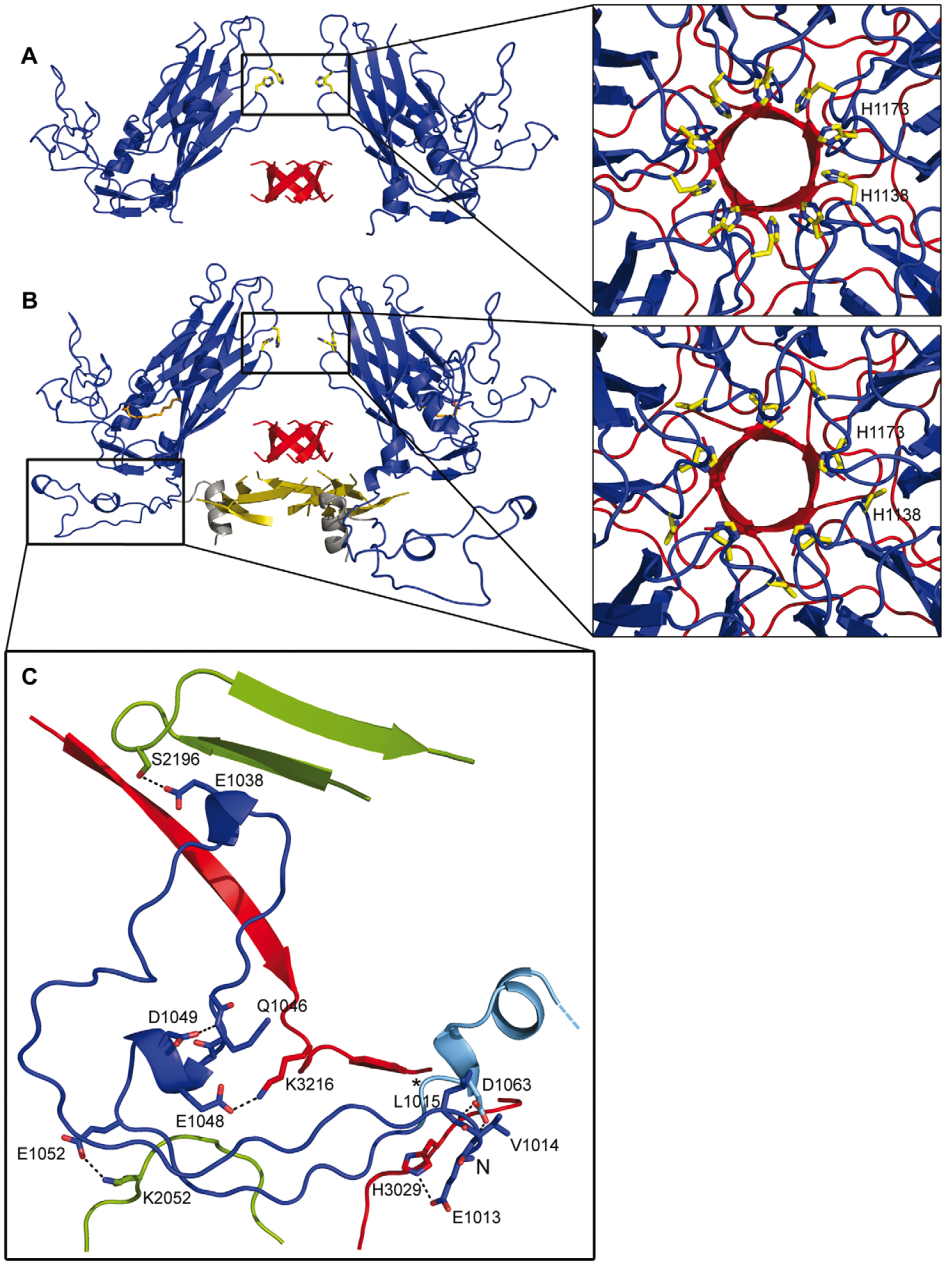
Changes around the five-fold channels. Lateral view of the five-fold axis in the 80S particle (A) and the native capsid (B) of HRV2. VP1 protein is shown in blue, VP3 in red and VP4 in yellow. For clarity, only two subunits of VP1 are displayed. In the native structure, the pocket factor is displayed as orange sticks and the N-terminal α-helix (residues 1001 to 1013, not present in the crystal structure of HRV2) has been modeled according to its position in the structure of HRV16 and is shown in grey. The close-ups at the right show a top view of the five-fold axes, with residues His1138 and His1173 from the five symmetry related VP1 subunits displayed as yellow sticks. (C) Close-up of the N-terminal region of VP1 from the HRV2 native structure, seen from the inside of the virion. VP1 is shown in blue, VP2 in green and VP3 in red; the first residue seen in the 80S density map (Arg1062) is indicated with an asterisk and from then on VP1 is colored in cyan. The side chains of the residues involved in acid-labile interactions stabilizing the conformation of the VP1 N-terminus are shown as sticks and explicitly labeled.

**VP3.** Superposition of the VP3 barrels shows that, as in VP2, the conformation of most of the VP3 loops is conserved between the native and the 80S structure. Only a rigid body displacement of the VP3 N-terminus with respect to the barrel is observed (Figure 1D). The N-terminal extension of VP3 runs nearly parallel to its position in VP3 in the native virion, at a distance of 3Å (measured as the Cα- Cα distance between Gly3001 residues of the superposed structures; Figure 1D). This deviation gradually diminishes until the chains converge at residue Asp3050, just after helix αA. The smooth decrease in distance allows maintenance of the intactness of the VP3 five-stranded ß-tube at the inner face of the five-fold symmetry axes while the ß-barrels of VP2 and VP3 are displaced with respect to VP1 (Figures 2E and 3 and Video S1). The VP3 C-terminus also appears slightly shifted between strands ßI and ßI' (residues from Gly3214 to Cys3219) and from Met3222 onwards. The VP3 GH loop is displaced by about 1–2 Å in its first half, adopting a new conformation between residues Ser3179 and Tyr3187 (with a maximum distance of 5.2 Å at position Arg3182; Figure 1D). Finally, the BC and FG loops at the three-fold axis and the EF loop, near the two-fold axis, also present local rearrangements compared to the native structure (Figure 1D).

Comparison of the overall positions of each VP subunit in the 80S structure to those corresponding to the native particle reveals that the relative positions of VP2 and VP3 with respect to each other are maintained. However, in the 80S particle these subunits are displaced with regard to the VP1 ß-barrel by between 4 and 6 Å towards the icosahedral three-fold axis. This is easily seen in the superposition of both protomers when the ß-barrel of VP1 is used as a reference (Figure 2E and Video S2). In fact, the displacement of VP2 and VP3 is parallel to the displacement seen at the αA helix and the C-terminal region of VP1 (Table S1). Moreover, there is a disulfide bridge between residues Cys2229 and Cys3120, at the VP2-VP3 interface (Figure 2E). This S-S bond, which was also present in the native HRV2 structure (PDB code: 1FPN), links VP2 and VP3, assisting the concerted shift of these subunits.

Thus, the hinge-type movement of VP1 and the parallel displacement of VP2 and VP3 facilitate the expansion of the protomer and, in consequence, of the outer limits of the pentamer (Figure S1). Together they led to a bigger, but thinner, empty capsid (Figure 1 and Video S1).

### The Hydrophobic Pocket

**Figure 4:**
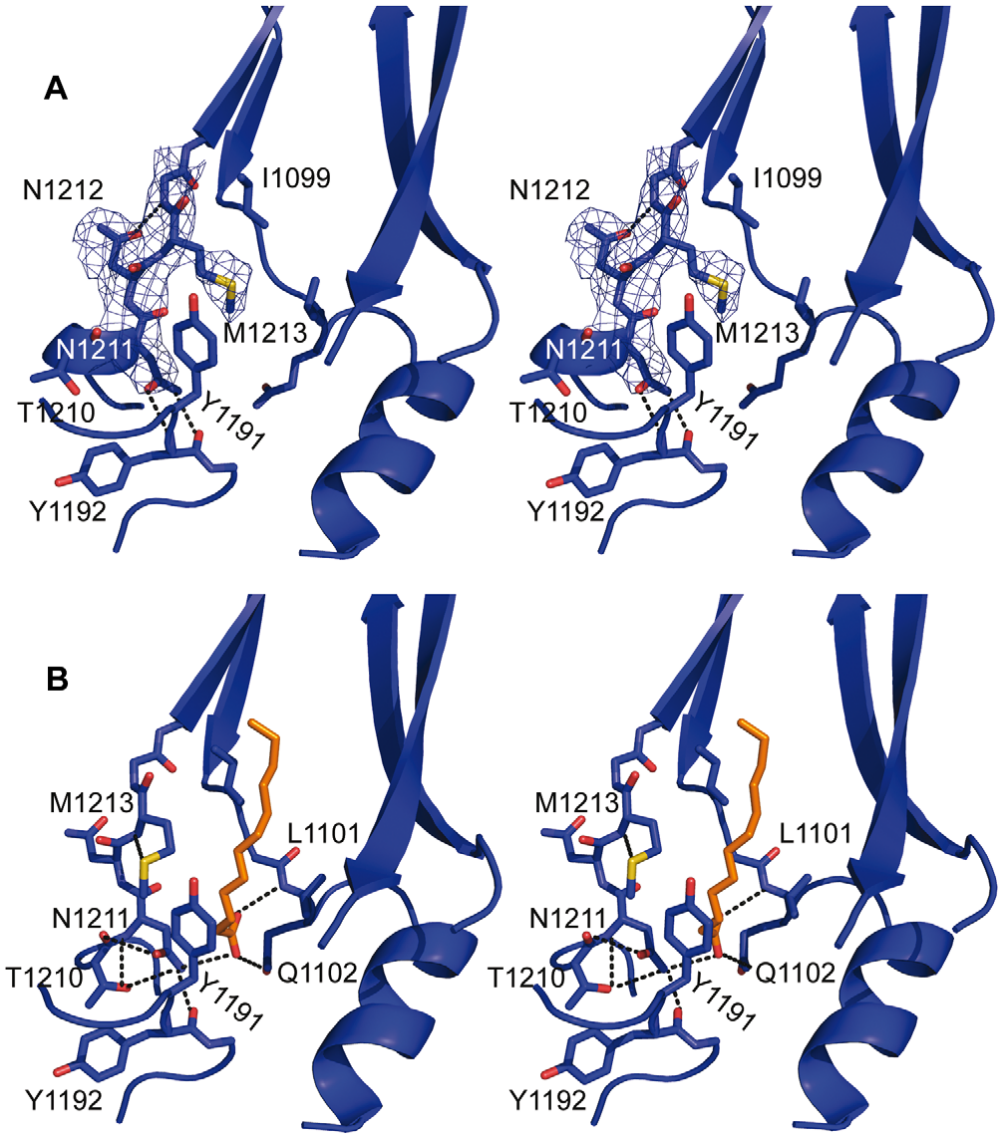
Structural changes in the hydrophobic pocket of VP1. Stereo views of the VP1 hydrophobic pocket in the empty capsid (A) and in the native HRV2 (B). For clarity, only some of the structural elements forming the pocket are shown. VP1 is represented as a blue cartoon and the relevant residues are shown as blue sticks and explicitly labeled. In (A), the averaged electron density map (contoured at 1.0 σ) is shown as a dark blue mesh around the GH loop. In (B), the pocket factor (a 12-carbon fatty acid) is depicted as orange sticks. The pocket cavity, in the 80S particle, adopts a closed conformation and the side chain of Met1213 has extended, crossing the binding site of the pocket factor. In the native capsid, the polar head group of the pocket factor makes hydrogen bonds with the main chain nitrogen from Leu1101 and Nɛ of Gln1102 (located in the CD loop) and with the Nγ of Asn1211 (GH loop, just before the H ß-strand). Conversely, in the 80S empty capsid, Asn1211 interacts with the main chain of Tyr1192 (located at the other end of the same GH loop, in the proximity of the hinge pivotal center). This allows Met1213 to occupy the position of the polar head group of the fatty acid, via interaction with residues Ile1099 and Tyr1191.

As stated above, the hinge-type movement of VP1 has its pivotal center in the region around Lys1243, which is located just below the ß-barrel, near the hydrophobic pocket in VP1. In the native virion, this pocket was filled with a density which was interpreted by the presence of a 12-carbon atoms long fatty acid (Figure 4B; [18]). In the structure of the 80S particle, the density within the pocket is absent and the cavity appears collapsed. The GH loop of VP1 has a conformation resembling the closed structure observed in HRV14 and HRV3 [15], [53], with the side chain of Met1213 in an extended conformation across the pocket factor binding site (Figure 4A).

### The Pore Below the Canyon

**Figure 5:**
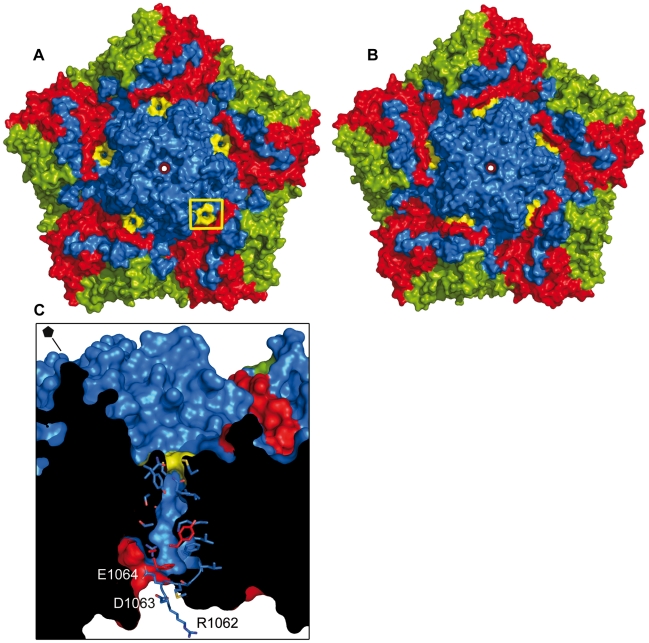
Formation of pores in the 80S empty particle. Surface representation of a pentamer from the 80S particle (A) and the native capsid (B), seen from the outside and along the five-fold symmetry axis. In both figures, VP1 is shown in blue, VP2 in green and VP3 in red. The residues located at the outer opening of the pores in the 80S structure (Met1104, Ala1105, Gln1106, Tyr1159 and Gln1162) and the corresponding residues in the native capsid are painted in yellow. In (A), the location of one pore is explicitly highlighted with a yellow square. (C) Lateral view of the pentamer region highlighted in (A), represented as surface and slabbed to show the pore that completely crosses the capsid from the canyon floor to the capsid interior. The side chains of residues located along the pore walls are shown as sticks in blue (VP1) and red (VP3). The N-terminus of the 80S VP1 structure is also shown as sticks and the residues located at the base of the pore (Arg1062, Asp1063 and Glu1064) are labeled. The side chain of residue Arg1062 has been modeled from the native capsid structure. For orientation purposes, the direction of the five-fold symmetry axis is indicated with a line and a black symbol at the upper-left corner.

Surprisingly, the 80S empty particle possesses pores or breaks crossing the capsid shell (Figure 5A). These pores, absent in the HRV2 native virions (Figure 5B), connect the canyon floor with the internal surface of the particle, close to residues Arg1062-Glu1068. This region corresponds to the first visible amino acids at the VP1 N-terminus and includes the acidic residues Asp1063 and Glu1064 (Figure 5C). The glutamic acid at position 1064 is strictly conserved in all HRVs except for the major group rhinoviruses HRV3 and HRV14, in which Asp1063 is replaced by Ser. (See alignment in [5]).

The opening of these pores at the canyon floor is formed by residues Met1104, Ala1105 and Glu1106 (within the αA helix) of one VP1 subunit and Tyr1159 and Gln1162 (in the αB helix) of the adjacent VP1. The walls of the channel are formed by three different regions contributed from two neighboring VP1 molecules (residues Ser1069, Phe1070, Leu1071, Arg1073 at the N-terminal loop, residues Ile1107 and Lys1110 from the αA helix and residues Ser1163, Gly1164 and Thr1165 from the αB helix) and by the C-terminus of VP3 (residues Ala3223, Asp3225).

### Re-organization of the Intra-pentamer Interfaces in the 80S Particle and the Channel at the Five-fold Axis

Capsid expansion implies significant rearrangements of the interactions at the interfaces between protomers, resulting in an overall loss of intra- and inter-protomer contacts in the HRV2 empty particle (Table S2 and Figure S2). A total of 197 interactions stabilized the intra-pentamer interfaces of the native HRV2 particles and only 95 of these contacts are conserved in the 80S particle. Twenty new contacts are formed, replacing some of the stabilizing interactions within the HRV2 80S pentamer. However, the overall contacting interfaces are weakened (Table S2). Some of the residues involved in the re-organization of the intra-pentamer contacts, in particular those located at the five-fold symmetry axes channel, are sensitive to a reduction in pH and conserved in the acid-labile rhinoviruses (Figure 3). The five-fold channel of the 80S particle appears slightly wider at its outer surface when compared to the equivalent structure in the virion. This is due to the structural rearrangements of loops BC, DE and HI in the 80S VP1 structure (Figures 1B, 3 and S1). The iris-type movement described in the cryo-EM structure of HRV2 80S particle [40] is generated by these loop changes (Figure 3 and S1A). In native HRV2 virions, this channel was surrounded by a ring of five symmetry-related aspartate residues (Asp1135, within the DE-loop), interacting with five solvent molecules surrounding a peak of density at the five-fold axis that was interpreted as stemming from a Ca^2+^ ion with partial occupancy [18]. The involvement of the DE loop of VP1 in the interaction with metal ions on the five-fold axis was found in the structures of all other rhinovirus analyzed. These cations were predicted to play a role in regulation of rhinovirus stability, although no conformational changes were observed in EGTA-treated virus structures [54]. In the 80S structure, the side chain of Asp1135 appears mostly disordered and no extra density is found to position any ion and/or solvent molecule in the region. Below the Asp1135 ring, there are five symmetry-related histidines (His1173, at the FG-loop). In the structure of the native virions, these histidine residues were bridged to each other through poorly ordered metal ions or solvent molecules [18]. In the 80S particle the DE loop is shifted towards the FG loop, thereby approaching a new histidine ring (built from His1138) to this area. In fact, this second group of histidines (His1138) appears to interact with His1173 of the neighboring VP1 subunit around the five-fold axis, closing the ring at this level (Figure 3A). The inner part of the five-fold channel is occupied by a ß-tube, of about 5.5 Å in diameter, formed by five symmetry-related N-termini of VP3 (residues from Gly3001 to Ser3010). In the 80S empty capsid, the VP3 ß-tube maintains not only its native conformation but also its exact position in the five-fold channel and most of the interactions with the surrounding residues as in the native virions (Figures 3 and S1B and Video S1). The intactness of the VP3 ß-tube between native and empty HRV2 capsids accounts for the displacement observed at the VP3 N-terminal extension when the ß-barrels are superimposed (Figure 1D).

### Re-organization of the Inter-pentamer Interfaces in the 80S Particle and Formation of a Channel at the Two-fold Axis

**Figure 6:**
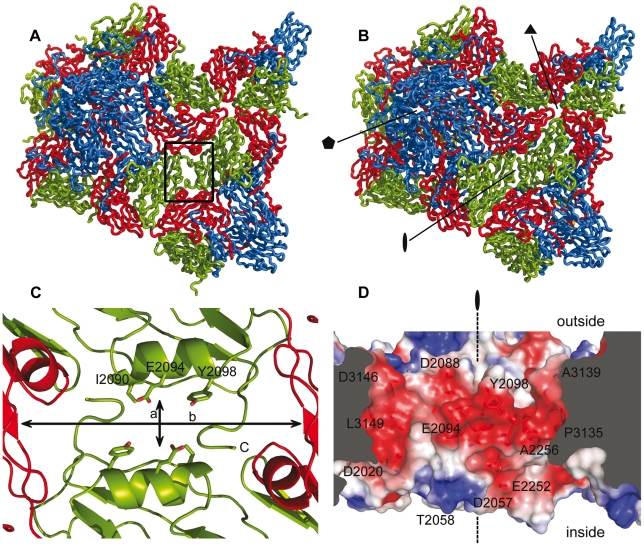
Opening of the two-fold channels in the 80S empty capsid. Contacts between pentamers along the two-fold and three-fold axes in the 80S empty particle (A) as compared to those present in the native HRV2 capsid (B). The capsid proteins VP1 (blue), VP2 (green) and VP3 (red) are depicted as ribbons. The position of the symmetry axes is indicated in (B). (C) Close-up view of the channel formed at the two-fold axes in the 80S particle, seen from the interior of the capsid. The VP2 and VP3 subunits surrounding the channel are represented as cartoons and the side chains of VP2 exposed at the interface are depicted as sticks. The lines crossing the cavity correspond to distances of 10.2 Å (a) and 30.5 Å (b). (D) Charge distribution on the walls of the channel at the two-fold axes. In order to show the surface of the longitudinal walls, a side view of the channel (perpendicular to the direction of the symmetry axis) has been slabbed. The surface is represented with the electrostatic potential, colored in blue and red for positive and negative charges, respectively. The relevant residues are shown as sticks and labeled. The direction of the two-fold symmetry axis is also indicated with a line and a black symbol.

In native HRV2 particles, a total of 99 interactions were established between pentamers. Only 36 of these contacts are conserved in the 80S particle, while 21 new contacts are formed, partially replacing the lost interactions (Table S2). About 30% of the 63 missing contacts involved the N-terminal end of VP1, which in the 80S structure is disordered up to position 1062 (Table S2 and Figure S2). In native HRV2 virions, the VP1 N-terminus was involved in extensive interactions with VP2 and VP3. Some of these contacts were mediated by acidic residues that are highly conserved in acid-labile rhinoviruses (Figure 3C). In addition, this region, together with the ß-hairpin of VP2 (strands A_1_–A_2_), participated in a crucial element stabilizing the pentamer interface: an extended antiparallel ß-sheet, comprising the VP2 ß-hairpin of one pentamer, sandwiched between the four-stranded CHEF sheet of the VP3 ß-barrel and the VP1 N-terminus of the adjacent pentamer. This pentamer assembly feature is found in all picornaviruses [55]. In the structure of the 80S empty capsid, the VP1 N-terminus is disordered and the ß-hairpin of VP2 is considerably displaced, making this ß-sheet shorter and, thus, weakening the inter-pentamer contacts. In addition to the re-organization of the inter-pentamer network, the αA helices of the two adjacent VP2 subunits, which were in close contact in the native capsid (Figure 6B and Table S2), become separated by 10Å in the 80S capsid (Figures 6A and C). Moreover, in the empty capsid, the AB loop of VP2 (residues from Ile2050 to Ser2059) is also displaced and appears to be highly flexible (see above). Altogether, these changes disrupt 13 interactions of the native capsid and produce a break in the interface between pentamers that crosses the capsid along the two-fold symmetry axes. The approximate size of this break in the HRV2 empty capsid is about 10×10 Å, having its length limited by the flexible most C-terminal residues of both VP2 subunits. Assuming the flexibility of the AB loop and VP2 C-terminus, the capsid break would then extend up to the ß-barrel of the adjacent VP3 subunits, allowing the expansion of the channel dimensions to approximately 25×10 Å (Figure 6C). Indeed, it is conceivable that it temporally expands even more during egress of the viral RNA.

## Discussion

During the viral life cycle, viral capsids must carry out several functions, some of which seem incompatible: self-assembly, packing and protection of the viral RNA, recognition of the appropriate host cell and delivery of the viral genome. Thus, the mechanical properties of viral capsids must change considerably to adapt to these tasks while preventing virion collapse. To solve these conflicting requirements, the picornaviral capsids are arranged as highly dynamic structures, where minimal changes in the protomer subunit lead to a global restructuration of the whole capsid and the gain of new properties. The activation energy barrier for the required conformational changes might be lowered *in vivo* by specific physiological factors, including virus binding to cell receptors, binding and unbinding metal ions or acidification in the endosomes. These factors will act as switches, adapting the metastable capsid to the precise requirements of the virion, according to its context and the stage of the virus life cycle [56]–[61].

Despite the wealth of structural information available on many aspects of the picornavirus capsid and its interactions with host cell receptors, some fundamental questions remain unanswered. In particular, little is known about the mechanism by which low pH triggers rhinovirus uncoating or about the dynamic process of genome release. The crystal structure of the HRV2 80S empty capsid and its comparison with the native virus shed light on the high resolution conformational changes that affect the capsid proteins during uncoating and unveil a number of residues susceptible to changes in their protonation state upon pH reduction that may play a key role in this process. Remarkably, these residues are conserved in the acid-labile rhinoviruses.

### The Hinge Movement in VP1 and the Capsid Expansion

Early studies in major group rhinoviruses suggested an allosteric competition between the pocket factor, binding below the canyon floor, and ICAM-1, binding to the outer face of the canyon floor [47]. According to this view, the expulsion of the pocket factor would allow stronger binding of ICAM-1 thus inducing a hinge movement near the pocket. This would lead to an opening at the five-fold axes, which was suggested to be the way for the RNA to exit the capsid [60].

In contrast, in the minor group virus HRV2, the receptor binding site does not overlap the pocket, and so LDLR does not enter into competition with the pocket factor [12], [14]. Instead, the viral particles travel with the lipoprotein receptor through clathrin-coated pits to the endosome, where viral uncoating is triggered by low pH [29], [30], [62]–[65]. Among other models, and by analogy with the major group rhinoviruses, it was thought that in minor group HRVs the RNA genome may also be externalized through the five-fold axis of the capsid, which would be connected to a pore on the endosomal membrane, formed by five N-termini of VP1 and five VP4 molecules [28].

The X-ray structure of the HRV2 80S empty capsid shows indeed a hinge movement around the VP1 pocket. However, and in contrast to previous predictions, this movement does not induce any widening of the icosahedral five-fold axis but facilitates the expansion of the capsid protomer, forcing a reorganization of the interfaces between protomers and resulting in the opening of new channels in the capsid core that enhance the permeabilization of the virion. These new breaches in the capsid could then act as gates for the externalization of the VP1 N-terminus and the extrusion of the viral RNA.

### The Externalization of the VP1 N-terminus

The 3D-structures of two representative members of the HRV-A species (HRV2 and HRV16) are available at resolutions higher than 2.6 Å [18], [19]. In the structure of native HRV2, the N-terminus of VP1 was disordered for the first twelve amino acids and weak density was observed for the positioning of Glu1013 and Val1014 [18]. The main chain amino group of Leu1015 is hydrogen-bonded to the side chain of Asp1063, located at the beginning of the α-helix and highly conserved in HRV-A and -B species (Figure 3C). In addition, the main chain of Asp1063 is hydrogen-bonded to the side chain of the strictly conserved Glu1013 residue (Figure 3C). The electron density for Glu1013 residues was poorly defined in the structure of native HRV2, but is clearly visible in HRV16, the only rhinovirus whose VP1 N-terminus could be traced entirely. In the HRV16 structure, Glu1013 is located at the C-terminus of an amphipathic α-helix which stacks on the outside of a ten-stranded β-barrel formed by five symmetry-related VP4 proteins, lying on the interior of the virus particle along the icosahedral five-fold axis [19] (Figure 3B). In the HRV16 virion the side chain of Glu1013 is hydrogen bonded with the main chain amino groups of Glu1063 and Glu1064 and with the side chain of the conserved His3029 of VP3 (Figure 3C). Additional conserved acidic residues in the region, Glu1048 and Glu1052, form polar interactions with Lys3217 and Lys2052 side chains of VP3 and VP2, respectively. All these interactions, which help to stabilize the conformation of the VP1 N-terminus at the capsid interior of native virions, are missing in the 80S structure, where the first amino acid seen in the electron density is Arg1062 (Figure 3A). The pore observed at the base of the canyon crossing the capsid appears to be a leftover of the exit site of the VP1 N-terminus (Figure 5C). The structure of the 80S particle shows that residues Asp1063 and Glu1064 are located at the internal surface opening of this pore. However, no ordered density was seen, neither within the pore nor in its vicinity, to build additional residues extending from Arg1062. The absence of ordered density could be explained because in our 80S particle, obtained by heating the virus at 56°C, the VP1 N-terminus was not extruded but it remains disordered in the capsid interior. In fact, the lack of hydrophobicity of the 80S particle is in favour of this [34]. Furthermore, the size of the pore, as seen in our 80S structure, appears too narrow for accommodating a polypeptide chain, although it might temporarily widen, in particular, on heating. It must be taken into account that the HRV2 80S empty capsid represents the relaxed state of the capsid after VP4 and the viral genome have been extruded.

The outer surface of this pore is surrounded by residues Met1104, Ala1105, Glu1106, Tyr1159 and Gln1162, located at the canyon floor, along the line connecting the three-fold and five-fold axes. This site is not totally coincident with the putative exit site of the PV VP1 N-terminus, as hypothesized on the basis of the cryo-EM reconstructions of 135S-derived particles [43], [48].

### The RNA Exit Site

The five-fold axis has been hypothesized as a port of exit for the RNA during infection since the first structures of mature rhinovirus and poliovirus were determined [15], [23]. The low-resolution cryo-EM structure of the HRV2 80S particle also suggested an iris-type of movement of VP1 that would open a passage of ∼10Å diameter through the five-fold axis [40]. In fact, the crystal structure of the 80S empty capsid shows a small widening at the outer surface of the five-fold channel (Figures 5A, 5B and S1A). However, the constriction observed at the five-fold interior, due to the presence of the VP3 β-tube, is maintained in both native virions and in the empty 80S particles (Figures 3A, 3B and S1B). The presence of this β-plug constriction makes RNA egress through at that channel very unlikely.

In contrast to the intactness of the protein organization at the 5-fold interior of the empty 80S particle, the reorganization of the inter-pentamer interactions due to capsid expansion induces a shift by about 5 Å to the VP2 helix αA0, located at the two-fold axis, contributing to the formation of the biggest holes that are seen in the structure of the 80S particle (Figure 6A and Video S1). The dimensions of these channels strongly suggest that they could serve as routes for the egress of the viral RNA out of the capsid. In fact, some recent observations also point to this conclusion: a structure of a poliovirus uncoating intermediate “caught in the act of RNA release”, determined by cryo-electron tomography [42] shows the externalization of the viral genome via one of the holes, close to the two-fold axis. Moreover, inspection of the electrostatic potential at the internal surface of the 80S two-fold axis (Figure 6D) showed that the channel is mostly electronegative but contains small electropositive regions at the inner and outer parts of the channel. The electronegative nature of the channel would facilitate the extrusion of the RNA molecule, traversing by floating away from the repulsive walls of the channel. Similar situations have been described in other proteins involved in translocation of nucleic acids [66], [67]. This electronegative surface is mainly contributed by the main chain oxygens of different amino acids (Figure 6D) and would remain essentially unchanged upon acidification.

## Materials and Methods

### Virus Propagation, Purification and Preparation of Empty HRV2 Capsids

HRV2 was grown in HeLa-H1 cells in suspension cultures and purified as previously described [12]. To obtain empty HRV2 particles, a suspension of HRV2 (0.03 mg/ml) in 50 mM Tris (pH 8.0) was heated to 56°C for 12 min. The quality of the particles obtained was checked by negative staining electron microscopy, showing a highly homogeneous material. Samples were then concentrated using Centricon 100K tubes (Millipore) to a final concentration of 3 mg/ml. The initial low concentration was found to be necessary to avoid aggregation and minimize the formation of A-particles.

### Crystallization and Data Collection

Crystals were obtained by the vapor diffusion method in hanging drops at room temperature by mixing equal volumes of 80S particles (3 mg/ml) and a reservoir solution containing 0.3 M to 0.6 M sodium acetate with a pH range between 6.5 and 8.0 and containing 5% glycerol (vol/vol) as an additive. Crystals were transferred to a cryo-protecting solution containing 20% glycerol in the crystallization buffer and incubated 1 min prior to cooling by immersion in liquid nitrogen. Ten data sets up to 3.0 Å resolution were collected from different crystals using synchrotron radiation at the ESRF, Grenoble, France (beamline ID23-1) and the SLS, PSI, Villigen, Switzerland (beamline X06DA), using a Mar-Mosaic 225 charge-coupled-device detector in both cases. Diffraction images were processed using MOSFLM [68] and internally scaled with SCALA [52] (Table 1).

### Structure Determination and Refinement

Crystals of HRV2 80S empty capsids, belonging to space group I222 and with unit cell parameters of a = 313.9Å, b = 357.8 Å c = 383.1 Å, were closely related with those of the native HRV2 [18] (a = 308.7 Å, b = 352.2 Å c = 380.5 Å), showing only a small enlargement in all three axes. In these I222 crystals, three icosahedral two-fold axes of the viral particle coincide with the three crystallographic two-fold axes, leaving ¼ of a virus particle (15 protomers) in the crystal asymmetric unit.

Initial phases were obtained after a rigid body fitting of the coordinates of the native HRV2 structure (PDB:1FPN) into the new unit cell, using the program CNS [69] with 15-fold non-crystallographic symmetry constraints. After 10 cycles of rigid body refinement, considering first the whole protomer (VP1, VP2, and VP3 proteins) as a unique body, plus 10 new cycles, considering VP1, VP2 and VP3 as independent bodies, the R factor decreased from 54.1% till 39.7% for data in the resolution shell 50.0−4.0 Å.

Similar results were obtained by using as an initial model the coordinates of the native HRV2 structure previously fitted into the 15 Å cryo-electron microscopy maps of the HRV2 80S particles by using URO [70] and the positioning of the new model into the HRV2 80S unit cell, followed by a rigid body refinement with CNS, considering VP1, VP2 and VP3 as independent bodies.

At this point a 2Fo-Fc electron density map was calculated to 4.0 Å resolution. Inspection of this initial map showed that the first 60 residues of VP1 were totally disordered and these amino acids were removed from the model prior to the calculation of the initial phases. Cycles of 15-fold non-crystallographic averaging and solvent flattening with the program DM [71] were used to refine and extend the initial phases from 6.0 to 3.0 Å resolution. The averaging and solvent masks used covered the whole asymmetric unit. The resulting density allowed us to determine most of the structural differences between native HRV2 and the 80S empty capsid and model rebuilding was started by using the graphic program Coot [72]. Refinement was performed with CNS with non-crystallographic symmetry constraints and bulk solvent correction, using data in the resolution shell 50.0−3.0 Å. New maps were calculated and iteratively improved by density modification cycles, using updated averaging and solvent masks, to a final correlation coefficient of 0.93. Iterative positional and temperature refinement using CNS was alternated with manual model rebuilding with Coot. The final refinement statistics are summarized in Table 1. The coordinates and structure factors have been deposited in the Protein Data Bank (code 3TN9).

### Illustrations

Figures were drawn and rendered with PyMol [73]. Electrostatic potential surfaces were calculated with the program APBS [74]. Animations were produced by using the Chimera visualization software package [75].

## Supporting Information

Figure S1 **Changes in the pentamer.** Outer (A) and inner (B) views of the 80S pentamer, displayed as a surface representation of its Cα. The color code corresponds to the displacement suffered for every residue when comparing its position in the native capsid and the 80S particle. The color scale, indicated as a bar, covers distances from 0 Å (dark blue) to 6 Å or more (red). Distances were calculated from a superposition of the native and the 80S pentamers, using the VP3 β-plug as a guide. In the inner view, the displacement of the different residues due to protomer expansion is proportional to their distance from the five-fold axis; the region surrounding the symmetry axis is mainly maintained (at least at the inner surface of the capsid), while the outer limits of the pentamer suffer the largest shifts.
(TIF)

Figure S2 **Changes in the inter-pentamer interfaces.** Ribbon representation of a native (A) and 80S (B) capsid protomer, viewed from the inside of the particle and with VP1, VP2, VP3 and VP4 proteins colored in blue, green, red and yellow, respectively. The secondary structure elements involved in interface interactions only in the native capsid or only in the 80S particle are displayed as cartoons in the corresponding structure and colored, respectively, in cyan and orange (C) Location of these changes in the capsid context. Inside view of three 80S pentamers related by a three-fold symmetry. The regions containing the biggest changes in the pentamer-pentamer interactions are displayed as cartoons and coloured as in (A) and (B).
(TIF)

Table S1 **The hinge movement in VP1 affects VP2 and VP3 positions.**
(DOC)

Table S2 **Interfaces of interaction in the native and the 80S capsids.**
(DOC)

Video S1 **The 80S HRV2 particle.** Rotational views of the outer surface organization and the capsid thickness of the native virion and the 80S particle. The changes at a two-fold and a five-fold symmetry axes are also shown. VP1 proteins are represented in blue, VP2 in green and VP3 in red.
(MOV)

Video S2 **Changes in the 80S protomer.** Comparison of the capsid protomer structures of the native virion and the 80S particle, highlighting the hinge movement of VP1 (in blue) and its effect on VP2 and VP3 (green and red, respectively).
(MOV)
