# Supplementary figures and images for: Insights into Minor Group Rhinovirus Uncoating: The X-ray Structure of the HRV2 Empty Capsid
Source: PLoS Pathog. 2012 Jan 5;8(1):e1002473. doi: 10.1371/journal.ppat.1002473 (PMC3252380; doi:10.1371/journal.ppat.1002473)

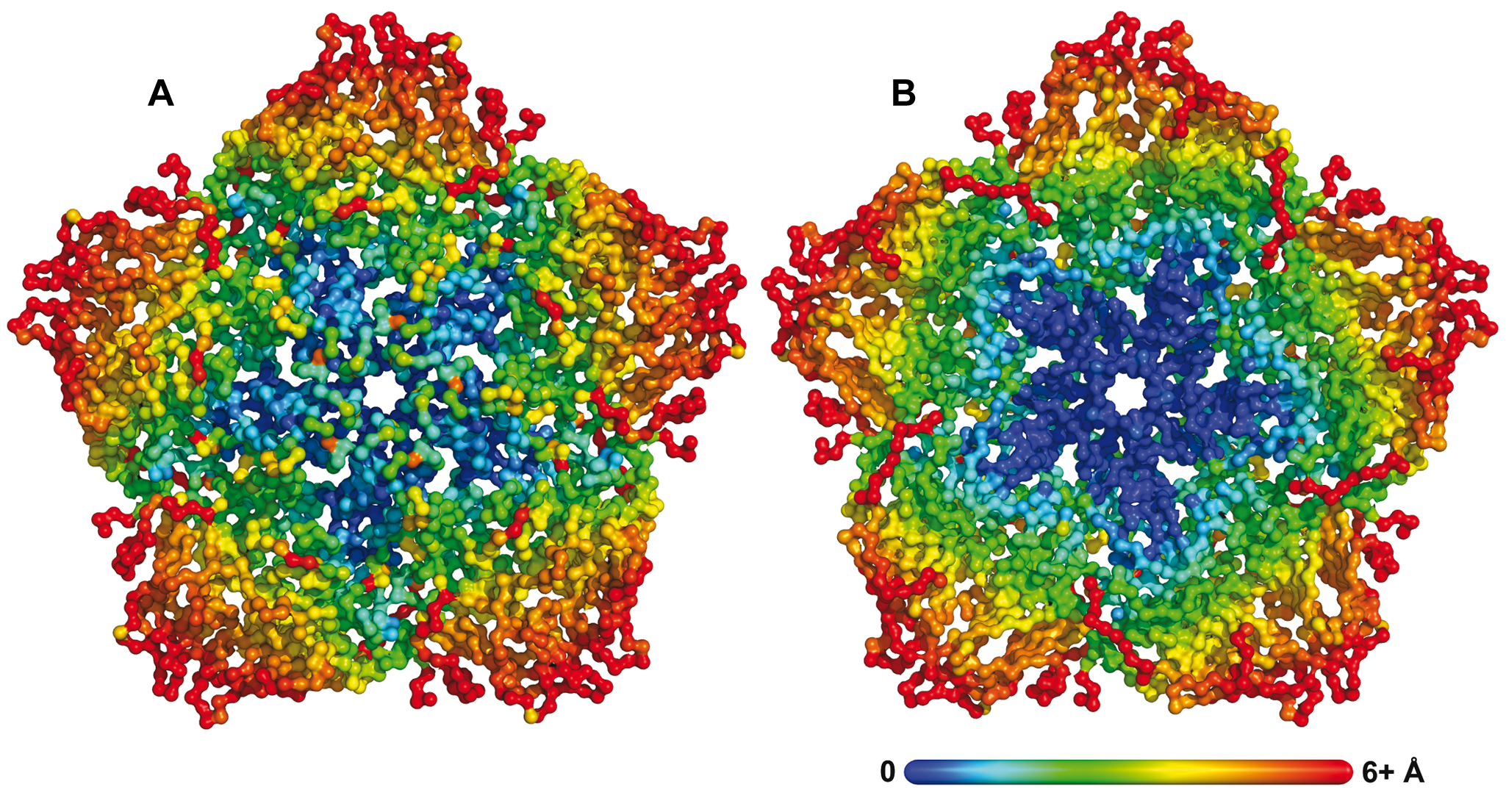

Supplement: Figure S1 — Changes in the pentamer. Outer (A) and inner (B) views of the 80S pentamer, displayed as a surface representation of its Cα. The color code corresponds to the displacement suffered for every residue when comparing its position in the native capsid and the 80S particle. The color scale, indicated as a bar, covers distances from 0 Å (dark blue) to 6 Å or more (red). Distances were calculated from a superposition of the native and the 80S pentamers, using the VP3 β-plug as a guide. In the inner view, the displacement of the different residues due to protomer expansion is proportional to their distance from the five-fold axis; the region surrounding the symmetry axis is mainly maintained (at least at the inner surface of the capsid), while the outer limits of the pentamer suffer the largest shifts. (TIF) [file ppat.1002473.s001.tif]

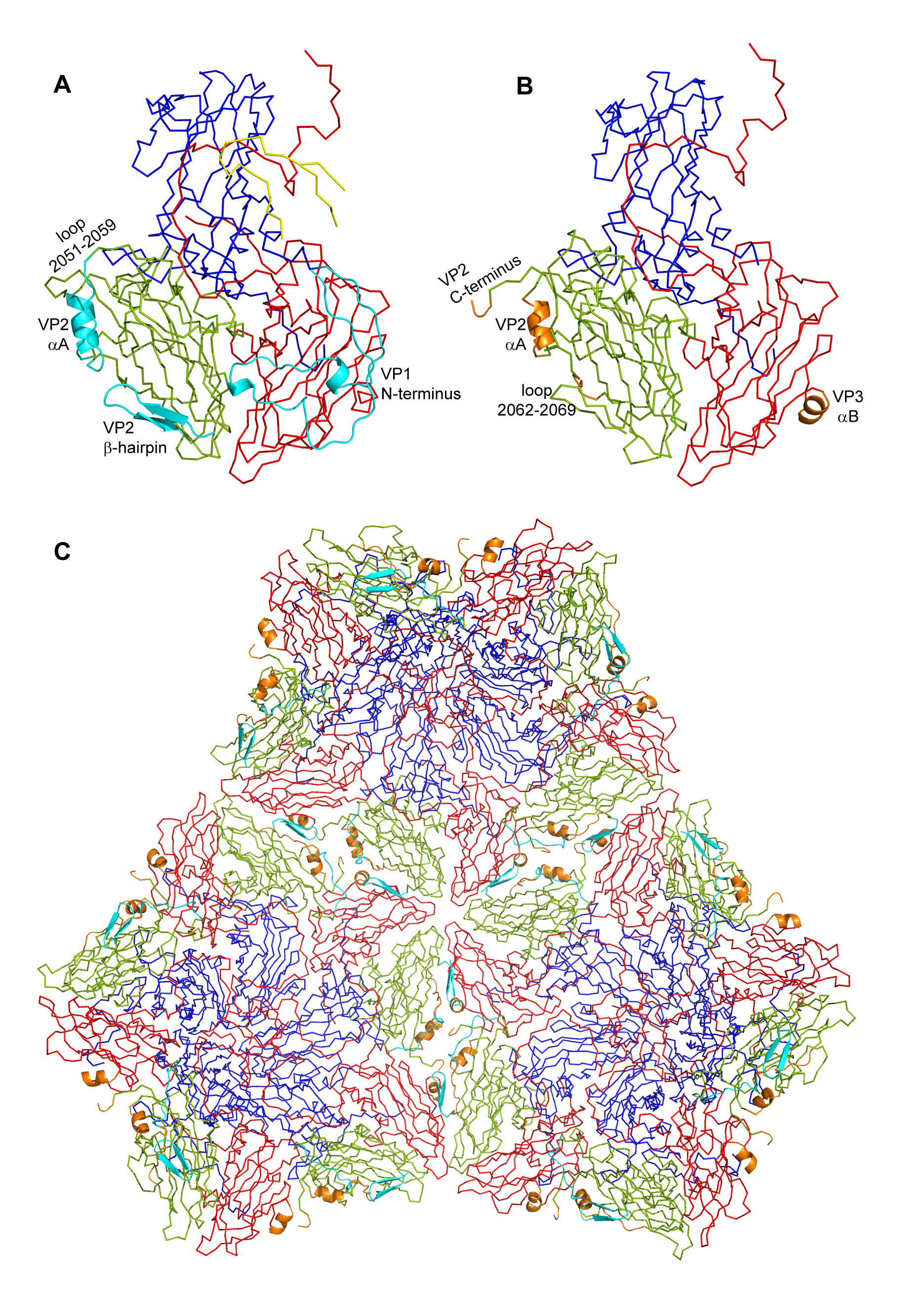

Supplement: Figure S2 — Changes in the inter-pentamer interfaces. Ribbon representation of a native (A) and 80S (B) capsid protomer, viewed from the inside of the particle and with VP1, VP2, VP3 and VP4 proteins colored in blue, green, red and yellow, respectively. The secondary structure elements involved in interface interactions only in the native capsid or only in the 80S particle are displayed as cartoons in the corresponding structure and colored, respectively, in cyan and orange (C) Location of these changes in the capsid context. Inside view of three 80S pentamers related by a three-fold symmetry. The regions containing the biggest changes in the pentamer-pentamer interactions are displayed as cartoons and coloured as in (A) and (B). (TIF) [file ppat.1002473.s002.tif]
