# Supplementary material for: Insights into Minor Group Rhinovirus Uncoating: The X-ray Structure of the HRV2 Empty Capsid
Source: PLoS Pathog. 2012 Jan 5;8(1):e1002473. doi: 10.1371/journal.ppat.1002473 (PMC3252380; doi:10.1371/journal.ppat.1002473)
Supplement: Table S1 — The hinge movement in VP1 affects VP2 and VP3 positions. (DOC) [file ppat.1002473.s003.doc]

**Table S1. The hinge movement in VP1 affects VP2 and VP3 positions.**

|  | **Residue** | **Region** | **ϕ_80S_ (º)** | **ϕ_150S_ (º)** | **Δϕ (º)** |
| --- | --- | --- | --- | --- | --- |
| VP1 | A1105 | αA helix | 42.70 | 36.47 | 6.23 |
|  | D1267 | C-terminal loop | 61.98 | 55.86 | 6.12 |
|  | A1283 | C-terminus | 85.72 | 81.35 | 4.37 |
| VP2 | W2071 | 3-fold axis | 110.52 | 104.18 | 6.34 |
|  | G2093 | αA helix, 2-fold axis | 117.21 | 112.85 | 4.36 |
|  | I2184 | pseudo-3-fold axis | 102.50 | 96.34 | 6.16 |
|  | I2203 | ß-barrel, near 5-fold axis | 140.59 | 134.33 | 6.26 |
|  | C2229 | disulfide bridge, VP2-VP3 interface | 105.17 | 99.13 | 6.04 |
| VP3 | F3005 | ß-plug at the 5-fold axis | 48.86 | 49.41 | -0.55 |
|  | S3035 | below VP1 ß-barrel | 142.99 | 141.55 | 1.44 |
|  | R3086 | outer capsid surface | 88.80 | 83.48 | 5.32 |
|  | V3127 | ß-barrel, 3-fold axis | 114.72 | 109.82 | 4.90 |
|  | L3149 | αA helix, near 2-fold axis | 104.35 | 98.82 | 5.53 |
|  | L3209 | ß-barrel, VP2-VP3 interface | 106.87 | 101.54 | 5.33 |

The angles between the C_α_ atoms of S1130, K1243 and a third residue were calculated, both for the native and the empty particles protomer. The difference between these two values is also given. Residue S1130 is located at the top of VP1 ß-barrel; residue K1243 is located in the I’ ß-strand, below the VP1 ß-barrel. The location of the variable residue is specified in the third column.
