# Supplementary material for: Insights into Minor Group Rhinovirus Uncoating: The X-ray Structure of the HRV2 Empty Capsid
Source: PLoS Pathog. 2012 Jan 5;8(1):e1002473. doi: 10.1371/journal.ppat.1002473 (PMC3252380; doi:10.1371/journal.ppat.1002473)
Supplement: Table S2 — Interfaces of interaction in the native and the 80S capsids. (DOC) [file ppat.1002473.s004.doc]

**Table S2. Interfaces of interaction in the native and the 80S capsids.**

|  |  | **Interactions** | | | | | **Buried Surface (Å2)** | | | **Association Energy (kcal/mol)** | | |
| --- | --- | --- | --- | --- | --- | --- | --- | --- | --- | --- | --- | --- |
|  |  | **Native** | **80S** | **Conserved** | **Lost** | **New** | **Native** | **80S** | **Difference** | **Native** | **80S** | **Difference** |
| **Intraprotomer** | VP1a-VP2a | 82 | 75 | 71 | 11 | 4 | 4178,7 | 3546 | 632,7 | -84,7 | -70,2 | -14,5 |
|  | VP1a-VP3a | 151 | 110 | 96 | 55 | 14 | 8620,8 | 6607,9 | 2012,9 | -188,8 | -142 | -46,8 |
|  | VP2a-VP3a | 66 | 62 | 57 | 9 | 5 | 3435,1 | 3482,9 | -47,8 | -68,9 | -68,5 | -0,4 |
|  | VP1a-VP4a | 15 | 0 | 0 | 15 | 0 | 899,6 | 0 | 899,6 | -14,2 | 0 | -14,2 |
|  | VP3a-VP4a | 14 | 0 | 0 | 14 | 0 | 818,4 | 0 | 818,4 | -11,5 | 0 | -11,5 |
|  | **Subtotal** | 328 | 247 | 224 | 104 | 23 | 17952,6 | 13636,8 | 4315,8 | -368,1 | -280,7 | -87,4 |
| **Intrapentamer** | VP1a-VP1b | 44 | 26 | 19 | 25 | 7 | 2275,6 | 1863,9 | 411,7 | -40,7 | -33,4 | -7,3 |
|  | VP1a-VP3b | 42 | 28 | 20 | 22 | 8 | 2874,1 | 2005 | 869,1 | -56,9 | -38,8 | -18,1 |
|  | VP2a-VP1b | 14 | 0 | 0 | 14 | 0 | 986 | 0 | 986 | -16,7 | 0 | -16,7 |
|  | VP2a-VP3b | 33 | 20 | 16 | 17 | 4 | 1523,7 | 1505,8 | 17,9 | -28,1 | -27,5 | -0,6 |
|  | VP3a-VP1b | 10 | 7 | 7 | 3 | 0 | 756 | 430,7 | 325,3 | -12,5 | -3,9 | -8,6 |
|  | VP3a-VP3b | 33 | 30 | 29 | 4 | 1 | 2069 | 1926,1 | 142,9 | -41,1 | -38 | -3,1 |
|  | VP3a-VP3c | 4 | 4 | 4 | 0 | 0 | 439,2 | 435,9 | 3,3 | -3,6 | -3,1 | -0,5 |
|  | VP3a-VP4b | 3 | 0 | 0 | 3 | 0 | 343,3 | 0 | 343,3 | -1,2 | 0 | -1,2 |
|  | VP3a-VP4c | 4 | 0 | 0 | 4 | 0 | n/c | 0 | n/c | n/c | 0 | n/c |
|  | VP4a-VP4b | 6 | 0 | 0 | 6 | 0 | 634 | 0 | 634 | -7,7 | 0 | -7,7 |
|  | VP4a-VP3b | 2 | 0 | 0 | 2 | 0 | n/c | 0 | n/c | n/c | 0 | n/c |
|  | VP4a-VP3c | 2 | 0 | 0 | 2 | 0 | n/c | 0 | n/c | n/c | 0 | n/c |
|  | **Subtotal** | 197 | 115 | 95 | 102 | 20 | 11900,9 | 8167,4 | 3733,5 | -208,5 | -144,7 | -63,8 |
| **Interpentamer** | VP1a-VP2g | 20 | 0 | 0 | 20 | 0 | 1293,9 | 0 | 1293,9 | -20,3 | 0 | -20,3 |
|  | VP2a-VP2m | 14 | 6 | 1 | 13 | 5 | 1495,7 | 681,5 | 814,2 | -26,5 | -11,4 | -15,1 |
|  | VP3a-VP2g | 63 | 49 | 33 | 30 | 16 | 2845,9 | 2415,5 | 430,4 | -54,6 | -47,4 | -7,2 |
|  | VP3a-VP3g | 2 | 2 | 2 | 0 | 0 | n/c | n/c | n/c | n/c | n/c | n/c |
|  | **Subtotal** | 99 | 57 | 36 | 63 | 21 | 5635,5 | 3097 | 2538,5 | -101,4 | -58,8 | -42,6 |
|  | **Total** | 624 | 419 | 355 | 269 | 64 | 35489 | 24901,2 | 10587,8 | -678 | -484,2 | -193,8 |

n/c : non-calculated values. This interfaces were not taken into acount in the total and differential calculations.

The number of unique residue pairs that contact at the subunit interface is listed for each interface in the native and the 80S particles. The number of common interactions in both capsids is stated in the fifth column. Sixth and seventh columns indicate the number of interactions only present in the native and in the 80S particles, respectively. The buried surface area for each interface of both capsids, as well as the difference between them, are stated in columns eigth to tenth. Association energies for each interface of both capsids, as well as the difference between them, are stated in columns eleventh to thirteenth. Interacting residue pairs, buried surface areas and association energies for each interface were calculated with VIPERdb tools [1].

The relative position of each protomer in the capsid is indicated with a letter after the capsid protein name (i.e. VP1a), as it appears in the following scheme:


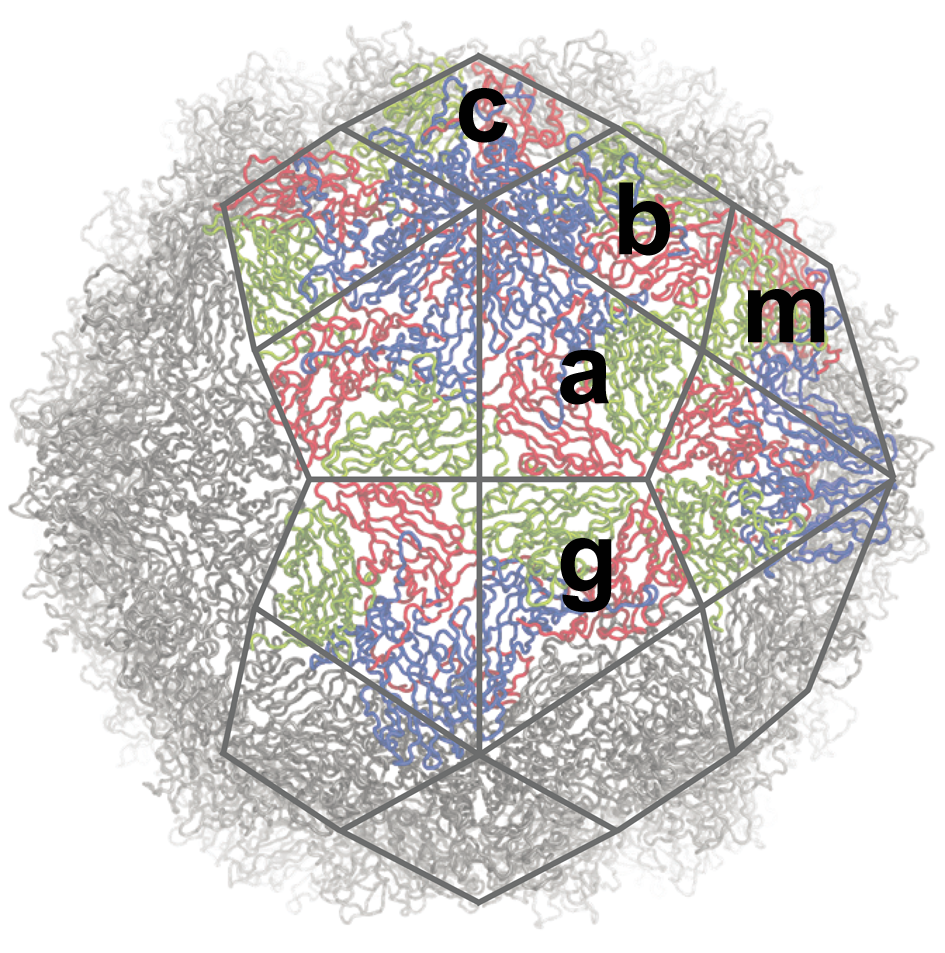


[1] Carrillo-Tripp M, Shepherd CM, Borelli IA, Venkataraman S, Lander G, et al. (2009) VIPERdb2: an enhanced and web API enabled relational database for structural virology. Nucleic Acids Res 37: D436-442.
